# Envy, Social Comparison, and Depression on Social Networking Sites: A Systematic Review

**DOI:** 10.3390/ejihpe13020027

**Published:** 2023-02-01

**Authors:** Fabio Carraturo, Tiziana Di Perna, Viviana Giannicola, Marco Alfonso Nacchia, Marco Pepe, Benedetta Muzii, Mario Bottone, Raffaele Sperandeo, Vincenzo Bochicchio, Nelson Mauro Maldonato, Cristiano Scandurra

**Affiliations:** 1 Department of Neurosciences, Reproductive Sciences and Dentistry, University of Naples Federico II, 80131 Napoli, Italy; 2 Department of Humanistic Studies, University of Naples Federico II, 80133 Napoli, Italy; 3 School of Integrated Gestalt Psychotherapy—SIPGI, 80058 Torre Annunziata, Italy; 4 Department of Humanistic Studies, University of Calabria, 87036 Cosenza, Italy

**Keywords:** social media, social networking sites, envy, upward social comparison, depression

## Abstract

This study aims to review the evidence for the reciprocal relationship between envy and social comparison (SC) on social networking sites (SNSs) and depression. We searched PsychINFO, PubMed, and Web of Science from January 2012 to November 2022, adhering to the Preferred Reporting Items for Systematic Reviews and Meta-Analyses guidelines. A total of 9 articles met our inclusion criteria. In all articles reviewed, a simple correlation was found between SC on SNSs, envy, and depression. Three cross-sectional studies successfully tested a model with envy as a mediator between SNSs and depression. The moderating role of additional variables such as self-efficacy, neuroticism, SC orientation, marital quality, and friendship type was also evident. The only two studies that were suitable to determine direction found that depression acted as a predictor rather than an outcome of SC and envy, and therefore depression might be a relevant risk factor for the negative emotional consequences of SNSs use.

## 1. Introduction

In an attempt to summarize several existing definitions, for the purposes of this review, we will broadly refer to social media (SM) or social networking sites (SNSs) as a group of websites or mobile internet-based applications that enable the formation of online communities through a virtual network and the sharing of user-created content such as information, ideas, opinions, messages, photos, and videos in a digital format [1,2,3]. Typically, these platforms require personalizing a profile within a limited system and creating a list of other users to interact with [4].

Today, an estimated 4 billion people use at least one SM platform [5], and according to some statistics, people worldwide spend an average of more than 2 h per day on SM [6]. In a relatively short period of time, SM has become a ubiquitous extension of daily social interactions for most teenagers and young adults and, to a lesser but growing extent, for other age groups [7]. Similar to other sudden societal changes occurring on a large scale, SM has raised growing concerns about its impact on mental health [8,9].

Extensive literature has begun to address the risks and potential negative effects of using SM [10]. Overall, as shown by meta-analytic evidence [11,12,13,14] and reviews of experimental studies [9,15], the existing literature suggests a small but negative impact of SM use on well-being, particularly depression. However, closer examination reveals that studies addressing this issue often yield contradictory or heterogeneous results [16,17], suggesting a shift in scientific attention to more specific mechanisms and psychological variables involved in the broad relationship between SM consumption and psychological well-being [18,19]. Given the conflicting results of previous studies that have attempted to examine the relationship between SNSs use and mental health, it is critical to identify the specific mechanisms underlying this relationship. One of the hypotheses put forward to better understand the harmful effects of SNSs assigns a prominent role to envy and social comparison [20,21,22]. Therefore, this systematic review aims to summarize studies that assess the relationships between envy, social comparison on SNSs, and depression.

### 1.1. Envy and Social Comparison on SNSs

Following Festinger’s theory [23] that people have an underlying motivation to evaluate themselves by referring to social information when objective parameters are not available, envy and social comparison are often considered to be closely intertwined [24,25,26]. Indeed, envy can be defined as a complex emotion [27] that encompasses a mixture of unpleasant and painful feelings such as inferiority, hostility, and bitterness [28] and arises as a contrastive reaction to an unflattering social comparison (i.e., the targets are perceived as advantageous or superior, and the comparators feel unable to narrow the gap between themselves and the target) [29]. As a result of such comparison, people feel that they lack the superior quality, performance, or possession of another [26].

For envy to occur, the comparison must occur in a self-relevant domain [28,30] and must target someone who shares salient characteristics with the envious person [31,32]. In other words, to elicit strong feelings of envy, the target of the comparison must appear similar, although seemingly superior. Usually, a distinction is made between benign and malignant envy based on the actions that trigger them. The former motivates the envious to improve their social standing to match the envied target, while the latter seeks to disparage the envied target or deprive it of the desired trait [33].

This premise is important for understanding the impact of using SM on well-being, as comparative information may matter more in the digital environment of SNSs than in offline interactions [34,35]. Design features that signal social status (such as “likes”) [36], the asynchronous nature of communication that provides ample time to filter one’s messages or comments, selection or editing practices to enhance one’s images [22,37], and the tendency to boast of successes and hide failures [17] are thought to induce a notable positive self-representation bias in SNSs [38]. As a result, one often encounters carefully crafted and overly embellished images of other users on SM [39,40], and high, possibly unrealistic, standards of comparison are easily applied [41]. Depending on the characteristics of each platform [26], users may also be tempted to interact primarily with friends and peers [42]. Consequently, by combining the elements of similarity, self-relevance, and perceived superiority of the comparison target, SM provides exceptionally fertile ground for the experience of envy [21].

Despite the attractiveness of this hypothesis, the complexity and limitations of the current evidence should not be overlooked [29,43], and it should be noted that depending on the type of envy elicited (e.g., malignant or benign envy), not only negative but also positive outcomes may be obtained [22,26]. Despite the need for caution in evaluating the present findings, a growing consensus is emerging about a relationship between social comparison and envy that negatively affects well-being [19,22,44].

### 1.2. Depression and its Connection to SNSs, Envy, and Social Comparison

Among the various domains of well-being impacted by the use of SM, depression has received particular attention, leading to the coining of the term “Facebook depression” in the media [45].

It has been suggested that people with depressive symptoms turn to SM for emotion regulation, for example, to avoid the anxiety of face-to-face interactions while still satisfying their need for contact [46,47]. However, their lack of self-esteem and tendency to compare themselves negatively with others may make depressed individuals particularly vulnerable to envy [21,48,49]. Indeed, people who meet the criteria for major depressive disorder have been shown to be more likely to engage in negative online behaviors such as social comparison and addictive use of SM [46]. Browsing the social content of Facebook News Feeds, which can trigger unflattering comparisons with idealized images of other users, has also been shown to be associated with lower self-esteem and depression [50], and several studies have demonstrated an association between depression and envy, both in an offline [51,52,53] and online setting [20,35,54].

Envy and social comparison have also been suggested as antecedents of depression in frequent users of SM [35,55,56].

### 1.3. The Current Study

The aim of our systematic review is to investigate whether the studies produced so far examining the relationship between envy and depression induced by social comparisons on SM paint a homogeneous picture and can help shed light on a limited but potentially crucial mechanism implicated in the nebulous, broader impact of media technologies on users’ well-being. We are aware of a few publications that have addressed the same questions [21,57,58], but they either did not precisely include the three variables we selected or did not use a systematic search strategy.

## 2. Materials and Methods

### 2.1. Search Strategy

The literature search strategy was conducted according to the Preferred Reporting Items for Systematic Reviews and Meta-Analyses (PRISMA) guidelines [59]. We assembled a wide range of search terms linked by logical operators: ((“social media” OR “social networking sites” OR “SNSs” OR “Instagram” OR “Facebook” OR “Tik Tok” OR “Twitter” OR “WeChat”) AND (“social comparison” OR “upward social comparison” OR “downward social comparison” OR “surveillance” OR “addict*”) AND (“envy”) AND (“depress*” OR “depressive disorder*” OR “depressive symptom*”)). The electronic databases searched for potentially eligible papers were PsycINFO, Medline/PubMed, and Web of Science. We then searched for additional relevant articles from other sources (e.g., Google Scholar).

### 2.2. Eligibility Criteria

We considered as eligible all papers written in English and published in peer-reviewed academic journals between 1 January 2012 and 10 November 2022. Reviews, meta-analyses, letters to the editor, books or book chapters, commentaries, abstracts only (congresses, etc.), and dissertations were excluded. Papers that examined the effects of SM use in general, rather than focusing specifically on the effects of engaging in SCs on SM, were excluded. The same criterion was applied to papers that focused only on psychological well-being and did not include a measure of depression.

### 2.3. Selection Methods

Two reviewers (FC and TDP) extracted the relevant data, excluded duplicates from the dataset, and independently reviewed titles and abstracts to assess article eligibility. Full-text articles were then examined for a final in-depth review. Disagreements among reviewers, which were few, were resolved through the involvement of the last author (CS).

## 3. Results

### 3.1. Identification of Eligible Studies

**Figure 1:**
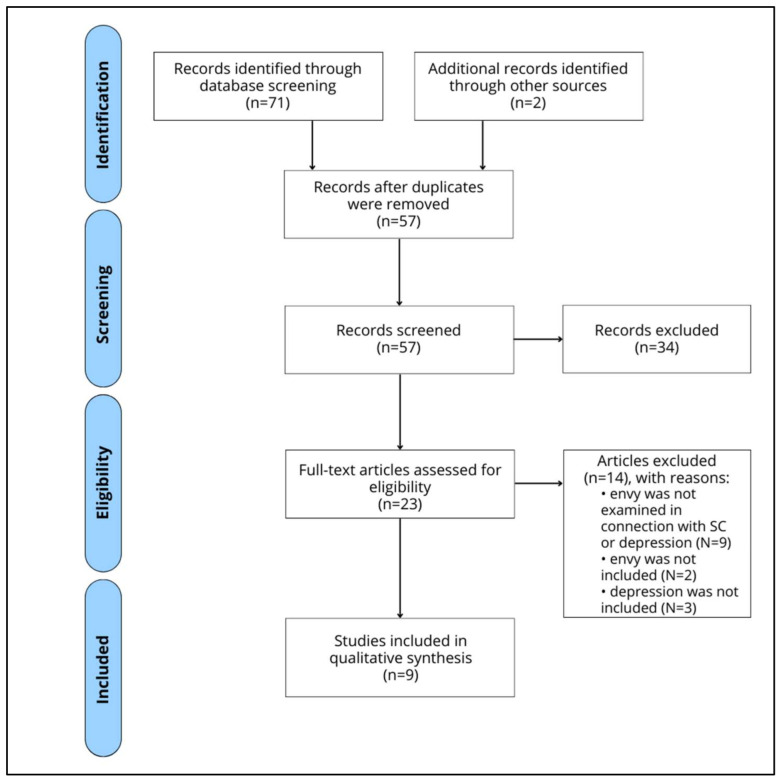
PRISMA flowchart of the systematic search.

Our search yielded 71 results, and only 2 records were added from other sources. After eliminating duplicates (16 duplicates), 57 titles and abstracts were screened, of which 34 were excluded for various reasons (e.g., the article was not relevant to the topic, the article was not in English, etc.). After a full-text review, 23 articles were retrieved and assessed for eligibility, of which 14 were excluded for the following reasons: (1) the article referred to envy but not to the association between upward social comparisons and depression (e.g., envy triggers material purchases) (*n* = 9); (2) the research examined depressive states resulting from upward social comparisons on SM without addressing envy (*n* = 2); (3) no direct measure of depression was reported (*n* = 3). A total of 9 studies were retained for the current systematic review. The screening process is schematically depicted in the PRISMA flow diagram (Figure 1). Table 1 summarizes key information on the selected articles.

### 3.2. Synthesis of the Studies

#### 3.2.1. Theoretical Framework

Three of the included studies [63,64,67] relied on Smith’s [25] typology of social comparison-based emotions, which categorizes emotional responses based on four dimensions. The first dimension is the direction of comparison, which can be either upward or downward depending on whether the evaluated target appears to be superior or inferior. The second dimension consists of the perceived control over the discrepancy that separates the comparator from the target. The intersection of these two dimensions allows for the classification of four types of emotions, while the dimensions of desirability, which refers to the positive or negative value of the observed outcomes of the comparison process, and the focus of attention, which can oscillate between self and other, are used to distinguish discrete emotions. Smith [25] asserts that in the case of upward social comparison, a positive/pleasant assimilative response is elicited when the comparator has high perceived control, evoking emotions such as admiration, optimism, and inspiration, i.e., upward assimilative emotions (UAE). If, on the other hand, an upward social comparison occurs, low perceived control leads to a negative/unpleasant contrastive response with emotions such as envy, shame/depression, and bitterness, i.e., upward contrastive emotions (UCE). If, on the other hand, a downward social comparison (DSC) occurs in which the target is viewed as inferior and its outcome is viewed as undesirable, high perceived control leads to a positive/pleasant contrastive response with emotions such as pride, contempt/disdain, and Schadenfreude (pleasure at another’s misfortune), i.e., downward contrastive emotions (DCE). Low perceived control in a downward social comparison instead leads to a negative/unpleasant assimilative reaction accompanied by feelings such as worry/anxiety, pity, or sympathy, i.e., downward assimilative emotions (DAE).

Park and Baek [63] also distinguished between two orientations of social comparison. Ability-based social comparison is usually associated with self-esteem and, thus, potentially, with feelings of inferiority. Opinion-based social comparison, on the other hand, is typically associated with evaluating whether one’s opinions are socially acceptable or factually correct and, thus, leads to beneficial confrontation rather than competition [69].

Scherr and colleagues [65] based their study on the Stress Generation Hypothesis [70], which states that depressed individuals are exposed to high levels of interpersonal stress due to dysfunctional emotion regulation and inadequate coping mechanisms and that the stress thus generated exacerbates the original depression and initiates a vicious cycle. Another theoretical model applied by Tandoc and colleagues [66] was Social Rank Theory, according to which people who strive for power or attractiveness and secure a higher position in the social hierarchy may be prone to depression if they perceive themselves as subordinate [71]. Finally, Li [61] combined Festinger’s social comparison theory [23] with Bandura’s social cognitive theory [72] to examine how self-efficacy can moderate the effects of disadvantageous social comparisons.

#### 3.2.2. Depression as an Antecedent of USC on SNSs

Appel and colleagues [60] compared depressed and non-depressed individuals in a quasi-experimental design. Participants were asked to compare themselves to the owner of a fictitious profile that was set as either attractive or unattractive, depending on the experimental condition. Depressed participants rated each profile owner as happier compared to themselves than non-depressed participants (*F* = 29.57, *p* < 0.001, *η*_p_^2^ = 0.26). The envy elicited by the attractive profile was higher than that elicited by the unattractive profile (*F* = 23.33, *p* < 0.001, *η*_p_^2^ = 0.22). In addition, an interaction was found between depression and envy (*F* = 5.58, *p* < 0.02, *η*_p_^2^ = 0.06), which significantly increased envy when depression was high and participants were confronted with an attractive profile. Similarly, Scherr and colleagues [65] showed that depression increased envy (*β* = 0.12, *p* < 0.001) and Facebook surveillance (*β* = 0.09, *p* < 0.05) over time, but the reverse effect was not observed.

#### 3.2.3. Negative Emotional Correlates and Outcomes of USC on SNSs

Chow and Whan [61] reported a positive correlation between neuroticism and social comparison on Facebook, envy, and depression (*r* ranging from 0.16 to 0.50, *p* < 0.01). In contrast to previous findings [73,74], friend network size was not significantly related to depressive symptoms or other variables in this study (*r* ranging from −0.02 to 0.05, *p* > 0.05). In the regression model of this study, depression was explained by gender, with female participants having lower depression scores (*β* = −0.15, *p* < 0.01), Facebook social comparison (*β* = 0.12, *p* < 0.05), neuroticism (*β* = 0.36, *p* < 0.01), envy (*β* = 0.29, *p* < 0.01), and an interaction time x neuroticism (*β* = 0.14, *p* < 0.01).

The articles studied also point to relevant associations with the cluster of unpleasant emotions that fall into the category of UCE, which includes envy and depression. Park and colleagues [64] found that social comparison was positively related to depression, envy, and other UCEs (*β* = 0.70, *p* < 0.001) but not to UAE (*β* = 0.07, *p* = 0.303). The study by Tosun and Kasdarma [67] showed that Passive Facebook Use was positively related to the frequency of USC (*β* = 0.48–0.54, *p* < 0.001, *R*^2^ = 0.23) and USC increased UCE (*β* = 0.46–0.28, *p* < 0.05, *R*^2^ = 0.08), which in turn increased the level of depression (*β* = 0.40–0.34, *p* < 0.05, *R*^2^ = 0.15). Park and Baek [63] found that social comparison, when focused on ability, was not only significantly related to envy and depression (UCE) (*r* = 0.53, *p* < 0.001) but also to worry and sympathy (DAE) (*r* = 0.13, *p* < 0.001).

#### 3.2.4. The Mediating Role of Envy

Three studies tested the role of envy as a mediator between USC and depression [59,63,65]. In particular, Li [62] found that envy partially mediated the association between social comparison on SNSs and depression. In this study, envy positively predicted depressive symptoms (*β* = 0.29, *p* < 0.001), whereas USC predicted envy (*β* = 0.32, *p* < 0.001), with a small remaining effect of USC on depression (*β* = 0.17, *p* < 0.001). Tandoc and colleagues [66] observed that envy mediated the relationship between Facebook surveillance and depression such that higher surveillance frequency was associated with higher depression scores when envy was triggered (*β* = 7.79, *p* < 0.001), whereas Facebook surveillance was associated with lower depression scores when envy was not triggered (*β* = −0.773, *p* < 0.05). Wang and colleagues [68] found that envy fully mediated the relationship between SC and depression. Indeed, envy was predictive of depression (*β* = 0.54, *p* < 0.001) and USC (*β* = 0.23, *p* < 0.001) with no significant effect of USC on depression.

#### 3.2.5. Protective Factors and Positive Emotional Outcomes of USC on SNSs

Appel and colleagues [60] observed that self-esteem correlated negatively with envy (*r* = −0.38, *p* = 0.001), suggesting that this may be a relevant factor in the predisposition to negative emotional experiences online. Self-efficacy also appears to be able to curb the frequency with which users engage in disadvantageous social comparisons (*r* = −0.12, *p* = 0.01), and it was also found to be negatively correlated with envy (*r* = −0.42, *p* = 0.001) and depression (*r* = −0.32, *p* = 0.001), moderating the relationship between these three variables [62]. Park and Baek [63] also found evidence for a potentially protective role of an opinion-based comparison orientation, which was more strongly related to positive UAE such as inspiration and optimism (*β* = 0.41, *p* < 0.001) than ability-based social comparison (*β* = −0.17, *p* < 0.05). In the model tested in this study, opinion-based social comparison also appeared to decrease UCE such as envy and depression (β = −0.22, *p* < 0.01). In the study by Tosun and Kasdarma [67], USC was more strongly associated with UAE (*β* = 0.46, *R*^2^ = 0.20, *p* < 0.001) than with UCE (*β* = 0.28, *R*^2^ = 0.08, *p* < 0.05) when the comparison target was a close friend. UAE, in turn, showed a negative association with depression, although contrary to the authors’ hypothesis, the negative association between UAE and depression was stronger when the comparison target was an acquaintance (*β* = −0.51, *p* < 0.001) than a close friend (*β* = −0.24, *p* < 0.05). Tosun and Kasdarma concluded that participants in their sample, belonging to a collectivist culture in which interpersonal bonds and social harmony are highly valued [75,76], may have felt pressured to show insincere positive feelings in the face of a close friend’s success and, thus, may not have experienced significant alleviation of possible depressive states. Marital quality also emerged as a potential moderator of the effect of social comparison on envy (*β* = −0.09, *p* < 0.05) and depression (*β* = −0.09, *p* < 0.01), such that a higher quality marital relationship acted as a protective factor by reducing the negative emotional experiences triggered by online social comparison [68].

#### 3.2.6. Behavioral Outcomes

The study by Park and colleagues [64] was the only one to examine how USC and social comparison-elicited emotions affect online behavior and found that UCE were associated with discontinuation intentions (*β* = 0.39, *p* < 0.001), posting malicious comments (*β* = 0.43, *p* < 0.001) and, surprisingly, posting positive comments (*β* = 0.14, *p* < 0.01). The authors suggested that posting positive comments in UCE, such as envy, may be an attempt to hide genuine resentful reactions that might be frowned upon in an online community.

## 4. Discussion

The purpose of this study was to examine the current state of the literature regarding a specific mechanism that may explain the complex relationship between SM and psychological well-being. This mechanism revolves around three main variables: (1) social comparison; (2) envy; (3) depression. Recent developments in media and communication technologies have been the subject of heated public and scientific debate since the early 2000s, leading to a large number of studies focusing on the well-being of SM users [9,18]. Nevertheless, only 9 articles met our inclusion criteria, and they were all published recently (from 2015). This suggests that the particular link between the three variables we describe is a relatively new area of research.

On the whole, all studies found a significant relationship between envy, depression, and SC [60,61,62,63,64,65,66,67,68]. However, it should be noted that the studies by Park and Baek [63] and Park and colleagues [64] considered envy and depression as facets of a single construct (UCE) and measured them with a single item each. In addition, Li [62], Tandoc and colleagues [66], Tosun and Kasdarma [67], and Wang and colleagues [68] have supported the hypothesis that envy acts as a mediator in the relationship between social comparison on SM and depression. At the same time, it should not be underestimated that the relationship between these three constructs is complex and highly nuanced and is also susceptible to the influence of additional personal or interpersonal variables such as self-efficacy [62], neuroticism [61], type of comparison orientation (ability-based vs. opinion-based social comparison) [63], type of comparison target (close friends vs. acquaintances) [67], and marital quality [68]. Another conclusion, important to put these results in perspective, can be drawn from the only two studies whose design allowed us to infer a causal relationship [60,65]. Namely, these two studies show that depression contributes to stronger feelings of envy and a higher tendency to social comparison, but not vice versa. Given the paucity of publications, any interpretation should be taken with caution, but we can at least consider the possibility that depression should be viewed as a predictor rather than as an outcome of SM use, as a risk factor that increases responses to social stimuli and susceptibility to online maladaptive behaviors that might otherwise be benign or harmless [65,77].

Some scholars in the past have advocated a clear stance against the consumption of SM on the assumption that it would have proven undeniably harmful to the mental health of new generations [78,79]. However, the general consensus seems to be moving toward less categorical positions, as a growing body of evidence suggests that the use of SM can promote positive emotional outcomes under the right conditions [22,26]. SM should not be treated as a monolithic entity in terms of its influence on psychological well-being and mental health [80,81]. In fact, two of the articles reviewed support this view. Tosun and Kasdarma [67] observed a higher prevalence of pleasant emotional states as a result of upward social comparison rather than envy or other UCE. Perhaps more interestingly, Tandoc and colleagues [66] found that Facebook surveillance, a variable similar to passive Facebook use that is generally considered harmful in the scientific literature [82,83], was associated with lower rather than higher levels of depression when envy was not elicited. In our view, these results support the call by Kross and colleagues [18] that future research should move away from general questions and attempt to explain under what conditions SM influences well-being with partially opposite downstream effects, taking into account individual and cultural differences.

Advances in this area can have important implications for interventions that target the design features of these virtual platforms. An illustrative example is the decision to hide the number of likes on Instagram, which is likely aimed at eliminating constant feedback, a feature of SM platforms that can have a negative impact on well-being [36,63]. Another solution, commented on by the authors of one of the studies reviewed [64], is the implementation of algorithms capable of detecting malicious comments that someone might write out of envy and prompting the user to reconsider their intentions.

The main limitation noted in the studies reviewed was that, with only 2 exceptions [60,65], all used cross-sectional studies; therefore, no firm conclusions can be drawn about the directionality of the above relationships. This methodological limitation also affects the validity of the studies that tested the mediating role of envy [60,66,67,68], as cross-sectional data can be misleading when analyzing longitudinal mediation processes that, by definition, unfold over time [84,85,86]. Another notable limitation is that 7 of the 9 studies under consideration were conducted on Facebook [60,61,62,63,65,66,67]. Future research should therefore expand the variety of SM platforms studied, as the specific affordances and design features of each website or app may foster different forms of user interaction [87]. As suggested by Kross and colleagues [18], norms and values that vary across different populations may promote different behaviors and emotions in online interactions with other users. However, our literature search revealed only one study [67] in which the authors used the cultural background of the sample to explain their findings. Additionally, the heavy reliance on self-report measures is not optimal. Such measures are prone to subjective bias and have been criticized, especially for reporting technology-related activities, as they tend to underestimate actual usage time [88,89]. Finally, we acknowledge that the lack of qualitative studies and the absence of expert interviews, as could be obtained by using the Delphi method [90], does not allow for data triangulation and limits the completeness of our analysis [91].

## 5. Conclusions

Like all radical technological innovations, SM has raised doubts and hopes, but it has not been easy to obtain sound knowledge about the actual effects. Notwithstanding the clear limitations of this fledgling field of scientific research, our review shows that some promising avenues of research have been opened. Beginning to understand the risk and protective factors that play a role in the mental health of users of SM, and especially under what conditions people are more likely to be exposed to stressful or otherwise harmful online experiences, could be a first step in raising users’ awareness and advising them on which behaviors to support and which are better avoided.

## Figures and Tables

**Table 1:** Review of the literature on the relationship between upward social comparison, envy, and depression.

Author	Year	Methodology	Sample	Platform	Findings
Appel et al. [60]	2015	Quasi-experimental	*N* = 89 M age = 27.45	Facebook	Positive correlation between envy, depression, self-reported inferiority, and low self-esteem. Depressed individuals feel more envious and inferior, particularly when faced with attractive Facebook profiles (significant interaction: depression x envy).
Chow and Whan [61]	2017	Online cross-sectional survey	*N* = 282 Age range = 18 to 73	Facebook	Positive correlation between neuroticism, envy, and depression. Gender, SC, envy, neuroticism, and an interaction time of Facebook use x neuroticism predict depression. The size of friend network has no significant correlations with other variables.
Li [62]	2018	Cross-sectional survey	*N* = 934 Age range = 16 to 21	Facebook	Positive correlation between SC and both envy and depression; negative correlation between self-efficacy and both envy and depression. Envy partially mediates the effect of SC on depression. The interaction SC x self-efficacy moderates the effect of SC on depression and the effect of SC on envy.
Park and Baek [63]	2018	Online cross-sectional survey	*N* = 331 M age = 32.05	Facebook	Positive correlation between ability-based SC and both UCE and DAE. Positive correlation between opinion-based SC and UAE. Envy and depression mediate the relationship between SC orientation and life satisfaction.
Park et al. [64]	2021	Online cross-sectional survey	*N* = 330 Age range = 20 to 49	Instagram	Positive correlation between USC and UCE but not UAE. Positive correlation between UCE and three behavioral outcomes: discontinuation intentions, posting of malicious comments, and posting of favorable comments. Only UCE and not UAE mediated the relationship between USC and all behavioral outcomes.
Scherr et al. [65]	2019	Longitudinal two-wave online panel-survey	*N* = 514 Age range = 14 to 79	Facebook	Positive correlation between Facebook surveillance, envy, and depression at both t1 and t2. No cross-lagged effect of envy or Facebook surveillance on depression. Significant cross-lagged effects of depression on both envy and Facebook surveillance.
Tandoc et al. [66]	2015	Online cross-sectional survey	*N* = 736 M age = 19	Facebook	Positive correlation between Facebook surveillance and envy. No significant correlation between frequency of Facebook use and depression. No significant correlation between the size of the friend network and envy. Envy mediates the relationship between Facebook surveillance and depression. The positive effect of Facebook surveillance on depression scores when envy is present; negative direct effect of Facebook surveillance on depression when envy is absent.
Tosun and Kasdarma [67]	2019	Cross-sectional survey	*N* = 319 M age = 20.72 (n1); 20.56 (n2)	Facebook	The type of comparison target (close friends vs. acquaintances) moderates the relationship between UAE and depression and the relationship between UCE and depression. UAE are more frequent than UCE; this difference is heightened when the comparison target is a close friend. Positive correlation between passive Facebook use and USC; positive correlation between USC and both UCE and UAE. Positive correlation between UCE and depression. Negative correlation between UAE and depression.
Wang et al. [68]	2020	Online cross-sectional survey	*N* = 514 Age range = 20 to 59	Non-specified SNSs	Positive correlation between SC, envy, and depression. Envy fully mediates the relationship between SC and depression. Marital quality moderates the effect of SC on depression and the effect of SC on envy.

Abbreviations: M = Mean; SC = social comparison; USC = upward social comparison; UAE = upward assimilative emotions; UCE = upward contrastive emotions; MSM = mobile social media.

## Data Availability

Data will be made available upon reasonable request to the corresponding author.
